# Relations between sleep quality and academic performance among Vietnamese high-school students

**DOI:** 10.3389/fpsyg.2026.1848504

**Published:** 2026-09-11

**Authors:** Trang Le, Bahr Weiss, Do Thi Linh Phuong, Ho Tuong Linh, Nguyen Xuan Tung

**Affiliations:** 1 Clinical Research Institute for Society, Psychology and Education (CRISP-E), VNU University of Education, Hanoi, Vietnam; 2 Faculty of Educational Sciences, VNU University of Education, Hanoi, Vietnam; 3 Department of Psychology and Human Development, Peabody College, Vanderbilt University, Nashville, TN, United States

**Keywords:** academic performance, insomnia, sleep duration, sleep quality, students, Vietnam

## Abstract

**Background:**

Impaired sleep quality and insomnia are important predictors of reduced life functioning. Among adolescents, an important domain affected by insomnia is academic achievement. Insomnia has multiple components, and understanding how different components are related to academic achievement is important, so interventions can be designed to most efficiently target insomnia components. The present study's purpose was to assess relations between different components of insomnia and academic functioning among high-school students in Vietnam, where academic achievement is highly valued and strongly emphasized for adolescents.

**Methods:**

*Six hundred* and thirty-nine high-school students from a more highly-developed, urban area (Hanoi) and a lesser-developed, rural area (Hung Yen) were cross-sectionally assessed. The analysis sample contained 541 students without missing data. Self-report questionnaires assessed (a) sleep quality and insomnia, (b) academic performance, and (c1) fatigue, and (c2) student motivation as potential explanatory variables.

**Results:**

Canonical correlation analyses indicated a significant (*p* < 0.05) canonical correlation (*r* = 0.20) between Insomnia and Academic Performance, with higher levels of insomnia symptoms associated with lower reported academic performance. Standardized canonical coefficients indicated that sleep quality components Sleep Duration, Day Dysfunction, and Medication Use had unique statistical relations to Academic Performance. The canonical variate for academic performance was primarily composed of Student-reported GPA. When Motivation and Fatigue were included as covariates, the canonical correlation between sleep quality and academic performance decreased by 25%, and became non-significant, suggesting these variables may function as explanatory variables of relations between sleep quality and academic performance. Moderator analyses indicated variation in results as a function of Grade but not Gender.

**Conclusions:**

Study results suggest that evaluation of Sleep Extension Therapy (which increases sleep duration) for improving academic performance in high school students in countries similar to Vietnam may be useful. Moderator analyses results suggest that for younger high school students, future longitudinal research should investigate whether insomnia interventions should emphasize a direct focus on reducing related functional impairment because of younger students' apparent higher sensitivity to functional impairment. For older high school students, future research should also assess whether it may be beneficial to emphasize increasing sleep duration, to directly reduce cognitive impairment associated with insomnia.

## 1 Introduction

In general, humans spend between one-quarter and one-third of their life in sleep, as sleep is an essential life function necessary for various restorative purposes for both physical and mental health (Mendelson, 2017). However, the value and utility of sleep varies as a function of the quality of the sleep. At low sleep quality, insomnia becomes a significant risk (American Academy of Sleep Medicine, 2023). Insomnia is a common mental health problem involving persistent difficulty initiating or maintaining sleep across the night, waking earlier than planned or desired, and/or non-restorative sleep, resulting in impaired life functioning (American Psychiatric Association, 2022; World Health Organization, 2019). Insomnia is a prevalent mental health condition, with a recent systematic review finding that the point prevalence for insomnia among adults across the world is over 16% (Benjafield et al., 2025). The prevalence among adolescents is believed to be even higher, with prevalence estimates for insomnia for this developmental group ranging from 20% to 25% or even higher (Uccella et al., 2023). Insomnia is linked to a wide range of associated features, including fatigue, difficulty concentrating, irritability, decreased energy, loss of motivation, and related problems, often directly leading to significant functional impairment in work and interpersonal domains (American Psychiatric Association, 2022; World Health Organization, 2019).

Among adolescents, research indicates that one particularly important area of life functioning strongly impacted by insomnia is academic performance. Hedin et al. (2020), for instance, found that among their sample of Swedish high-school students, insomnia was associated with a 75% increase in the risk of academic course failure, with 12.8% of students without insomnia reporting course failure in comparison to 22.4% of students with insomnia (*p* < 0.0001). Academic achievement is a critical area of life functioning for adolescents, as it is a major predictor of future life success across a wide range of domains including physical and mental health, and social and economic success and well-being (OECD, 2017).

“Insomnia” has several underlying components (Manber and Carney, 2015). These include (1) sleep onset latency (how long it takes the individual to fall asleep), (2) sleep disturbance (the extent to which your sleep is disrupted by needing to go to the bathroom, by breathing problems, etc.), (3) the amount of time lost during such wakings, (4) sleep efficiency (the percentage of time that one is actually asleep while attempting to sleep), and several other such factors. These different components ultimately result in (5) sleep duration, the overall amount of sleep that an individual obtains nightly. There is some evidence that these different components of insomnia may have different effects on life functioning. For instance, it has been found that increased sleep latency is specifically associated with an increased focus on negative stimuli (Nota and Coles, 2018) whereas decreased sleep efficiency has been found to be related to impairment in cognitive recall (Rai and Sundarakumar, 2024).

Understanding how these different components of insomnia may affect the outcome of interest (in the present case, academic functioning) is critical in order to most efficiently structure interventions (Manber and Carney, 2015). For instance, Sleep Restriction Therapy focuses on increasing sleep efficiency by limiting the time the individual remains in bed if they are not asleep (Wei et al., 2025). This increases sleep efficiency, but at the cost of decreasing total sleep duration. In contrast, Sleep Extension Therapy involves gradually extending the time spent in bed, increasing sleep duration but at least initially decreasing sleep efficiency (Arnal et al., 2015). Thus, understanding which components of insomnia to first target for a specific outcome (e.g., academic achievement) is crucial.

The present study focused on relations of different components of insomnia to academic performance, among high-school students in the Southeast Asian nation of Vietnam. Vietnam has a strong cultural focus and family emphasis on academic achievement, with a child's academic success being seen as an obligation to the family and a primary moral duty of the child (Park et al., 2021). However, to the best of our knowledge there have been no published research studies investigating relations between insomnia and academic performance in Vietnam. Thus, understanding how prevalent health conditions such as insomnia are related to academic performance and which components of insomnia may be best to first target is critical. In addition, such information may be useful for public health purposes, to help families understand the importance of providing treatment and support for a child with serious insomnia problems, by documenting the link between insomnia and the valued domain of academic achievement.

Two important factors that may underlie relations between insomnia and academic performance are motivation and fatigue. One factor in academic (and most other domains') success is motivation, which directly influences the behaviors that lead to academic success (Diseth, 2025). It has also been found that fatigue plays an important role in academic success (Puah et al., 2024), in part through its effects on motivation. Given that motivation and fatigue are both directly influenced by insomnia (Walker, 2017), the present study assessed these two factors as potential links between insomnia and academic achievement.

## 2 Methods

### 2.1 Participants and sampling frame

The goal of the sampling frame was to provide a sample of Vietnamese high-school students representing a range of socio-economic conditions in Vietnam to enhance generalizability. Hanoi and Hung Yen provinces were purposively selected to include (respectively) (a) a more highly developed, urban area, as well as (b) a lesser developed, more rural area. Within each region, two high-schools participated in the project, and within each school two classrooms within Grade 10 and two classrooms within Grade 11 participated in the study. In Vietnam, high-school consists of grades 10, 11, and 12. The present study did not include grade 12 as students in this grade are focused on college entrance exams and applications, and generally do not have time for non-academic activities (Weiss et al., 2024). A total of 750 participants were contacted, with 639 providing student assent and parent consent, for an 85% participation rate. Among these, 85% had no missing data, yielding a final analysis sample of 541 students.

### 2.2 Measure translation

Measures for which there was not an extant Vietnamese version were translated and back translated by a bilingual team of psychologists in Vietnam and the US using standard procedures to maintain the semantic, technical, and conceptual content of the measure. In this process, we followed a consensus approach (Byrne, 2016; Hambleton, 2005) to translation rather than strict translation-back translation. In strict translation-back translations, translators often may make literal translations of items that back translate well to the original wording but fail to capture critical nuanced meanings in the translation. This failure may not be identified in the back translation since both the translation and back-translation are similar literal translations (Byrne, 2016; Hambleton, 2005). A Vietnamese clinical psychologist fluent in English, and a US clinical psychologist fluent in Vietnamese served as the primary translating team. After the initial translation/back translation, measures were reviewed by the full research team. Translations were adjusted based on their feedback. The team is highly experienced in measure adaptation and translation and is the official translator and distributor for several well-respected assessment instruments in Vietnam (e.g., the WISC-IV; Dang et al., 2011; the Child Behavior Checklist; Dang et al., 2017).

### 2.3 Measures

Measures' internal consistency was assessed using the omega statistic (Dunn et al., 2014; Vaske et al., 2017), with a minimum acceptable internal consistency value of 0.50 (George and Mallery, 2021; Thompson et al., 2010).

#### 2.3.1 Demographics

Basic background information including gender and grade was assessed in a demographic questionnaire.

#### 2.3.2 Sleep quality and insomnia symptoms

Sleep quality and insomnia symptoms were assessed using the Vietnamese version of the *Pittsburgh Sleep Quality Index* (PSQI) (Buysse et al., 1989; Nguyen et al., 2024). The PSQI is a widely used self-report questionnaire that rates sleep quality and insomnia symptoms over the last month. Although the word “insomnia” is not used directly in the scale itself, the PSQI has been validated for use in assessing insomnia and sleep disturbance. For instance, in the initial publication reporting the PSQI (Buysse et al., 1989), the PSQI correctly identified 84% of patients with *Disorders of Initiating and Maintaining Sleep*. In Backhaus et al. (2002), the PSQI showed a specificity of 84% and sensitivity of 99% for distinguishing between insomnia patients and controls. Mollayeva et al. (2016) conducted a systematic review and meta-analysis of the PSQI and concluded that the PSQI showed “strong reliability and validity” in regard to assessment of sleep dysfunction and insomnia. Thus, although the word “insomnia” is not used directly in the scale itself, the PSQI is considered a validated measure for assessing insomnia and sleep disturbance.

The PSQI includes 24 items grouped into 10 sections. For the Vietnamese version, Section #10 (Do you have a bed partner or roommate?) was dropped because this item was seen as inappropriate for adolescents living with their parents. The first four questions assess the time the individual typically goes to bed, how long it takes to fall asleep, what time the individual typically gets up in the morning, and how many hours of actual sleep the person gets. The PSQI items are scored into seven dimensions: (1) Subjective Sleep Quality, (2) Sleep Latency (how long it typically takes to fall asleep) (3) Sleep Efficiency (the proportion of your time in bed asleep), (4) Sleep Disturbance (the extent to which one's sleep is impaired because of needing to go to the bathroom, breathing problems, etc.), (5) Sleep Duration, (6) Use of Sleep Medication (prescription and/or non-prescription), and (7) Day Dysfunction (e.g., trouble staying awake during the day; lacking enthusiasm to get things done). Components are scored on a 0 to 3 scale, with 0 being minimal problems and 3 being severe problems. Thus, higher scores on the PSQI indicate lower sleep quality and higher insomnia. Omega internal consistency was not assessed for the individual PSQI scores since they are based on one or two items, but the omega for the combined PSQI items was 0.79.

#### 2.3.3 Academic performance

Participants provided their current GPA score (Self-reported GPA) and a subjective rating of their performance regarding their (a) overall academic performance as well as for (b) mathematics, (c) literature, and (d) foreign language, which are generally seen as three of the most important subjects in Vietnam (Dang et al., 2022; Dương et al., 2021). Student reported their grade point average for each of the above domains, on the Vietnamese academic scale which ranges from 1 (unacceptable) to 10 (outstanding). For the current study, students' grades were categorized into four categories from 1 to 4, with 4 being the best grade and 1 being the worst grade: (4) greater than or equal to 8.0; (3) 6.5 to less than 8.0; (2) 5.0 to less than 6.5; (1) below 5.0. For the subjective ratings, for each of the four domains participants indicated whether they thought that their performance was 4 = (Dương et al., 2021) Excellent; 3 = Good; 2 = Okay; or 1 = Unsatisfactory. The different academic domains were combined into summary scores, Student-reported GPA and Subjective Academic Performance. Omega internal consistency was 0.88 for Student-reported GPA and 0.87 for Subjective Academic Performance.

#### 2.3.4 Motivation

Participants completed the Vietnamese version of the Academic Motivation Scale (AMS; Nguyen and Nguyen, 2019). The two broadband Internal Motivation and External Motivation subscales were used. Internal Motivation involves motivation that comes directly from the participant's own interests and values (Ryan and Deci, 2017) (e.g., *How much do you enjoy learning new things at school*). External Motivation is motivation that comes from outside the individual, such as rewards, pressures, or expectations imposed by others or the environment (Ryan and Deci, 2017), in the case of the AMS from the participant's family and teachers (e.g., *How much do your parents praise or reward you for being successful in school*). Items are rated on a 0 (Not at all) to 4 (A great deal) Likert scale. The omega internal consistency was 0.81 for Internal Motivation and 0.72 for External Motivation.

#### 2.3.5 Fatigue

In the present study, the total score from the Chalder Fatigue Scale (CFS; Chalder et al., 1993) was used to assess fatigue. The Vietnamese version of this measure, which contains eight items, was used in the present study. Items focus on indicators of fatigue (e.g., *Do you feel sleepy or tired*) as well as consequences of fatigue (e.g., *Do you need to rest frequently*). Items are rated on a 0 (Never) to 3 (Most or all of the time) Likert scale. The omega internal consistency for the CFS was 0.90.

### 2.4 Procedures

For student recruitment, in each participating class a research assistant introduced the study, answered any questions about the study, and provided interested students with a study description and consent form for the parents. Only students who returned a signed parental consent form and also provided their own signed assent participated in the study. Data collection was conducted in a group meeting in a classroom during a free period, with research assistants providing an overview of the questionnaire, response categories, etc. Participants then completed the questionnaire, which took about 30 min. Each student who participated received a small gift of school supplies worth the equivalent of approximately 1 USD. This study was reviewed and approved by the Institutional Review Board at VNU University of Education – Hanoi, Vietnam, with Approval #25.04/HDDD-DHGD. All participants who provided data provided their assent as minors, after their parents provided their legal consent.

### 2.5 Data analysis plan

SAS 9.4 was used for the various analyses described below. Question 1 pertained to the overall relation between students' sleep quality (as assessed by the Pittsburgh Sleep Quality Inventory) and their academic performance (as assessed by students' report of their grade point averages as well as their subjective ratings of their performance). Because the objective of these analyses was to assess in an exploratory manner (i.e., without pre-specifying the structure or number of dimensions) the (a) structure of relations between the multi-dimensional domains of insomnia symptoms and academic performance, and (b) within these domains identify individual variables underlying these relations, canonical correlation analysis (CCA) was used. These analyses were conducted within the SAS Proc Cancorr procedure. CCA is designed to examine the shared multivariate association between two sets of correlated variables, without pre-specifying the structure of the relations (e.g., the number of factors). It achieves this by deriving linear combinations (canonical variates) that maximize the correlation between the two domains, and thus was seen as appropriate for the current analyses. Question 2 pertained to the unique (i.e., non-overlapping) relations of the various sleep quality components (as assessed by the PSQI) with academic performance. This question was addressed within the canonical correlation analysis, using the standardized canonical coefficients from Proc Cancorr which assess the unique relations between the variables and their canonical variate. In these analyses, the 0.40 cutoff for substantive interpretation of standardized canonical coefficient was used (Stevens, 2009). Question 3 evaluated the unique effects of the potential explanatory variables (motivation, fatigue) by entering them into the canonical correlation analysis as covariates. Their potential as statistical explanatory variables was assessed by determining whether their inclusion in the analyses resulted in relations between the PSQI and Academic Performance becoming (1) non-significant, (2) marginally significant, or (3) remaining significant (Ledermann et al., 2025).

Question 4 pertained to potential moderators of the statistically unique relations between sleep quality and academic performance identified in Question 2. These exploratory analyses were conducted in a multivariate general linear models analysis, parallel to the canonical correlation, with the PSQI variables as the independent variables and the two academic performance variables as the dependent variables. Gender and grade were evaluated as moderators by including their interaction terms in separate models. The gender and grade factors were dummy-coded by SAS, using the standard 1, 2 dummy-coding in Proc GLM for 1 df categorical variables.

### 2.6 Data assumption analyses

#### 2.6.1 Linearity of relations

To evaluate linearity of the relation between the two canonical variates (Insomnia and Academic Performance), a non-parametric lack-of-fit test was conducted by comparing the (a) fit of an OLS linear model (b) with the fit of a non-linear, more flexible LOESS model, using a lack-of-fit comparison between the restricted linear model and the more flexible non-parametric LOESS model (Fox and Weisberg, 2018; Hastie and Tibshirani, 2017). LOESS was selected because it makes minimal assumptions about the functional form of the relation and can detect departures from linearity across the full range of the data. In the first step, a baseline parametric OLS linear model was produced from the canonical variate scores to specify the restricted (linear) relation. In the second step, an unrestricted non-parametric locally-weighted smoothing (LOESS) model was produced using an Akaike Information Criterion (AICC) optimization procedure. The effective degrees of freedom consumed by the non-parametric fit were derived from the trace of the smoother matrix. The incremental reduction in residual sum of squares between the linear model and the LOESS model assesses the non-linearity of the canonical variate relation (Harrell, 2015). A non-significant F test indicates that any non-linearity in the observed data likely represents random sampling variation rather than systematic non-linearity. Section 3.2.1 below reports results of these analyses.

#### 2.6.2 Multicollinearity

Multicollinearity within each of the two domains (insomnia, academic achievement) was assessed using regression-based diagnostics. In these analyses, within each of the two domains (insomnia, academic achievement), a series of analyses were conducted with each variable within the domains serving as the dependent variable, with all other variables as the independent variables. Each dependent variable was analyzed in a separate analysis. Thus, for the insomnia domain with seven variables, seven regression models were estimated; for the academic achievement domain with two variables, two regression models were estimated. For each model, the Variance Inflation (VIF) and Tolerance statistics (James et al., 2021; Kim, 2019) were calculated to assess multicollinearity. Cut-offs for possible multi-collinearity problems were (a) for the Variance Inflation Factors (VIF) statistic VIF > 5.0 and (b) for the Tolerances statistic < 0.20 (James et al., 2021; Kim, 2019). Section 3.2.2 below reports results of these analyses.

#### 2.6.3 Multivariate outliers

Evaluation for potential multivariate outliers across the combined set of variables was conducted using the Mahalanobis Distance approach (Stevens, 2009; Tabachnick and Fidell, 2018). Mahalanobis Distance is a multivariate metric used to detect multivariate outliers. In contrast to simple univariate methods, it evaluates a data point's distance from the center of a multidimensional distribution (rather than the mean of a univariate distribution of a single variable) while accounting for both the variance of individual variables and the covariance among them. An observation can fall within normal ranges on every individual variable yet be a multivariate outlier because its combination of values breaks the expected covariance structure. Section 3.2.3 below reports results of these analyses.

#### 2.6.4 Multivariate normality

The Mardia test (Johnson and Wichern, 2019; Mardia, 1970) for multivariate normality was used in the present study. This test is a multivariate extension of univariate normality tests that evaluates whether the joint distribution of a set of variables conforms to a multivariate normal distribution (Johnson and Wichern, 2019; Mardia, 1970). It produces two complementary statistics: multivariate skewness and multivariate kurtosis. The multivariate skewness statistic assesses the degree of asymmetry in the joint distribution by examining third-order moments based on Mahalanobis distances among all pairs of observations. The multivariate kurtosis statistic evaluates the degree to which observations are more or less concentrated around the multivariate centroid than expected under multivariate normality. The multivariate kurtosis statistic is based on the fourth powers of the Mahalanobis distances. Both statistics have known asymptotic sampling distributions, thus allowing for formal significance tests of departures from multivariate normality. Because the conventional significance tests for canonical correlation analysis rely on multivariate normality, a non-parametric permutation procedure (Winkler et al., 2020) was prespecified to evaluate the statistical significance of the canonical correlations if the multivariate normality assumption was not met.

## 3 Results

### 3.1 Missing data

Eighty-five percent of participants had no missing data in the primary study variables and were included in analyses. Thirteen percent had missing data for one variable, 2% had missing data for two variables, and one participant had missing data for three variables. To evaluate potential patterns of missingness, we examined bivariate and multivariate relations between the number of missing data points and primary study variables (Gender, Grade, self-reported GPA, subjective academic achievement, and PSQI Total). Relations between the number of missing data points and other study variables were non-significant: (a) total PSQI *F*_(1, 637)_ = 0.85, *p* > 0.35; (b) student-reported GPA *F*_(1, 632)_ = 0.11, *p* > 0.70; (c) subjective academic performance *F*_(1, 631)_ = 0.69, *p* > 0.40; (d) Grade *F*_(1, 630)_ = 1.26, *p* > 0.25; (d) Gender *F*_(1, 631)_ = 0.91, *p* > 0.30. We also tested the overall model, with the Number of Missing Data Points as the dependent variable and each of the five variables above (Gender, Grade, self-reported GPA, subjective academic achievement, PSQI_Total) as a separate independent variable in the model. The overall model was non-significant (*F*_[5, 620]_ = 1.07, *p* > 0.35) as well all five of the independent variables: Gender *F*_(1, 620)_ = 0.97, *p* > 0.33; Grade *F*_(1, 620)_ = 1.27, *p* > 0.26; self-reported GPA *F*_(1, 620)_ = 0.02, *p* > 0.40; subjective academic achievement *F*_(1, 620)_ = 1.93, *p* > 0.15; PSQI_Total *F*_(1, 620)_ = 1.17, *p* > 0.25.

Although these non-significant associations offer preliminary evidence that missingness was not strongly systematically related to the observed variables, missingness mechanisms (e.g., MAR vs. Missing Not at Random [MNAR]) cannot be definitively established or empirically proven (Enders, 2022). Consequently, complete-case analysis was retained for simplicity, but potential bias stemming from unidentified missingness mechanisms remains a limitation of the study.

### 3.2 Descriptive statistics

Table 1 reports the means and standard deviations for the primary study variables, insomnia (PSQI) and academic performance (Student-reported GPA, and Subjective Academic Performance). Table 1 also reports the proportion of participants showing any level of the insomnia component of the PSQI. For instance, 0.68 of participants reported some level of Sleep Latency problem (i.e., typically taking longer than 15 min to fall asleep) whereas 0.08 reported Medication Use to help with their insomnia (see Table 1).

**Table 1:** Mean and standard deviations for primary study variables.

PSQI sub-scale	Mean (SD)^a^	Proportion with > 0 level of symptom^b^
Overall subjective sleep quality	1.08 (0.71)	0.82
Sleep disturbance	1.13 (0.60)	0.89
Sleep duration	0.82 (0.99)	0.50
Sleep efficiency	0.49 (0.91)	0.28
Sleep latency	1.10 (0.96)	0.68
Day dysfunction	0.98 (1.00)	0.59
Medication use	0.13 (0.50)	0.08
Academic performance^c^	Mean (Std)	
Objective rating (GPA)	2.97 (0.71)	
Subjective rating	2.86 (0.71)	

^a^All PSQI sub-scales are on a 0 to 3 scale, with higher scores indicating higher levels of the symptom or functional impairment.

^b^Proportion of participants who scored greater than 0.0 on the PSQI sub-scale; i.e., who reported some level of this component of insomnia.

^c^Academic performance was rated on a 1 to 4 scale, with higher scores indicating better academic performance.

### 3.3 Data assumptions results

#### 3.3.1 Linearity of relations

The assumption of linearity between the canonical variates was evaluated by comparing a restricted ordinary least squares (OLS) linear model with an unrestricted non-parametric LOESS model. The OLS model accounted for 4.05% of the covariance between the canonical variates, with *F*_(1, 539)_ = 22.78, *p* < 0.001 and a residual sum of squares of 518.10. The LOESS model selected the maximum smoothing parameter (1.00) using the corrected Akaike Information Criterion (AICC = 0.969), indicating that the optimal nonparametric fit was essentially equivalent to a linear fit. The LOESS model produced minimal reduction in the residual sum of squares (RSS = 517.84, from the OLS RSS = 518.10), representing an improvement of 0.26 residual sum-of-squares units (0.05%) relative to the linear model. After accounting for the additional degrees of freedom consumed by the LOESS smoother (Trace[L] = 2.48), the approximate lack-of-fit test was not statistically significant, with F(0.48, 538.52) = 0.57, *p* = 0.333. These findings indicate that the more flexible non-parametric model did not provide a significantly better fit than the linear model. Accordingly, the assumption of linearity was considered to be met.

#### 3.3.2 Multicollinearity

For the Variance Inflation Factor (VIF), the range of scores for the nine models was from 1.00 to 1.42. Given VIF > 5.0 as the standard cutoff for multicollinearity (James et al., 2021; Kim, 2019; Marcoulides and Raykov, 2019), results were interpreted to indicate no evidence of multicollinearity. For the Tolerance statistic, the range across the nine models was 0.70 to 1.00. Given Tolerance < 0.20 as the standard cutoff for multicollinearity (James et al., 2021; Kim, 2019; Marcoulides and Raykov, 2019), the Tolerance statistic also was interpreted to indicate no evidence of multicollinearity. Thus, there was no evidence of multicollinearity within either variable set.

#### 3.3.3 Multivariate outliers

Using the Mahalanobis distance approach (Stevens, 2009; Tabachnick and Fidell, 2018), within the analysis sample of 541 observations, six observations were identified as multivariate outliers. These observations' data were manually reviewed to assess validity (e.g., presence of fixed pattern responses; responses outside the actual range of the variable). Because no evidence suggested that these observations reflected data-entry errors, invalid response patterns, or impossible values, they were considered valid observations from the target population and were retained for analyses.

#### 3.3.4 Multivariate normality

The Mardia test (Johnson and Wichern, 2019; Mardia, 1970) for multivariate normality was used to assess multivariate normality in the present study. Results indicated Mardia multivariate skewness = 2,378 with *p* < 0.001, and Mardia multivariate kurtosis = 27.26 with *p* < 0.0001. Consequently, because the inferential significance tests associated with canonical correlation analysis assume multivariate normality, a non-parametric permutation test was used (see Section 3.4, below) to evaluate the statistical significance of the canonical correlations (Good, 2010; Pesarin and Salmaso, 2025). However, it is important to note that although violations of multivariate normality can affect the sampling distributions used for conventional significance testing, the canonical correlation coefficients themselves are valid descriptive estimates of the observed linear association between the two variable sets, regardless of normality or non-normality of the data. Thus, the standard canonical correlation analysis results were used for the canonical correlation parameter estimates.

Unlike conventional parametric significance tests, the permutation procedure does not assume any distributional form for the data (Pesarin and Salmaso, 2025). Instead, it empirically estimates the null distribution of the test statistic by repeatedly randomizing the correspondence between the two sets of variables while preserving the covariance structure within each variable set, across 9,999 iterations, generating an empirical null distribution of Wilks' lambda (Pesarin and Salmaso, 2025). The permutation *p*-value was calculated as the proportion of permuted datasets that produced a Wilks' lambda equal to or smaller than the observed value, with the standard correction of adding 1.0 to both the numerator and denominator to avoid zero *p*-values (Phipson and Smyth, 2010).

### 3.4 Question 1

A canonical correlation analysis was conducted to examine the overall relations between sleep quality and academic performance, using SAS Proc Cancorr. The first canonical correlation was R_C1_ = 0.20, and the second canonical correlation was R_C2_ = 0.11. Under the multivariate normal-theory significance test, the first canonical correlation was significant with Wilks' Lamda = 0.948, *F*_(14, 1064)_ = 2.07, *p* < 0.0113 whereas the second canonical correlation was non-significant (see Table 2). However, because the results of Mardia's test suggested that the assumption of multivariate normality was violated (see Section 3.3.4, above), the non-parametric permutation test involving 9,999 random permutations was conducted to evaluate the statistical significance of Wilks' lambda for the canonical correlations. Across the 9,999 permuted datasets, 107 produced a Wilks' lambda less than or equal to the observed value, yielding a permutation *p*-value of.0108. Similarly, 88 permuted datasets produced a first canonical correlation greater than or equal to the observed first canonical correlation, yielding a first-root permutation *p*-value of.0089. Thus, the non-parametric permutation analysis indicated that the first canonical function was statistically significant despite the violation of multivariate normality.

**Table 2:** Canonical correlation analysis results.

	Canonical function 1		Canonical function 2	
	R_c_ = 0.20^*^		R_c_ = 0.11	
PSQI sub-scale	CV.A.1SC^a^	CV.A.1Co	CV.A.2SC^a^	CV.A2.Co
Overall subjective sleep quality	−0.01	0.35	0.15	−0.06
Sleep disturbance	0.05	0.37	−0.08	0.03
Sleep duration	0.68	0.59	−0.24	0.04
Sleep efficiency	−0.33	0.01	0.41	−0.33
Sleep latency	−0.24	−0.02	0.03	−0.10
Day dysfunction	0.47	0.61	−0.60	0.43
Medication use	0.50	0.59	0.81	−0.72
Academic performance	CV.B.1SC	CV.B.1Co	CV.B.2SC	CV.B2.Co
Student-reported GPA	−0.74	−0.96	1.01	−0.29
Subjective rating	−0.36	−0.81	−1.20	0.59

^*^The nonparametric permutation test produced p = 0.0108.

CV.A.1SC = Standardized Coefficient for Canonical Variate A.1;

CV.A.1Co = Correlation coefficient for Canonical Variate A.1;

CV.A.2SC = Standardized Coefficient for Canonical Variate A.2;

CV.A.2Co = Correlation coefficient for Canonical Variate A.2;

CV.B.1SC = Standardized Coefficient for Canonical Variate B.1;

CV.B.1Co = Correlation coefficient for Canonical Variate B.1;

CV.B.2SC = Standardized Coefficient for Canonical Variate B.2;

CV.B.2Co = Correlation coefficient for Canonical Variate B.2.

^a^ Standardized canonical coefficients.

Coefficients greater than or equal to 0.40, the cut-off for indicating a unique contribution to the canonical variate, are bolded.

Overall then there was a relatively small but statistically significant relation between sleep quality (as assessed by the PSQI) and academic performance. Loadings for the Academic Performance canonical variates were negative, indicating that the higher the level of insomnia symptoms (i.e., higher scores on the PSQI), the lower the reported academic performance.

### 3.5 Question 2

For Question #2, to identify the unique contributions on academic performance of the various components of sleep quality from the PSQI, the standardized canonical coefficients were inspected for the significant relation, canonical correlation #1. As Table 2 indicates, for the significant Canonical Function 1, there were three PSQI variables with coefficients greater than or equal 0.40, the cut-off used to indicate that the variable made a conceptually meaningful contribution to the canonical variate (Stevens, 2009). In regard to the Academic Performance canonical variate, only Student-reported GPA scored above the 0.40 criterion, with a negative coefficient (−0.74) indicating that the higher the levels of insomnia reported on the PSQI, the lower the reported grade point average by the student (see Table 2).

### 3.6 Question 3

Question #3 assessed the effects of inclusion of Motivation and Fatigue as covariates in the canonical correlation analyses, testing them as potential explanatory variables of relations between sleep quality and academic performance. This analysis was conducted in three steps (see Table 3). Step A was the canonical correlation for the relation between the PSQI and Academic Performance without covariates (as reported above in Question 1). Step B included Motivation and Fatigue as covariates, each in separate models. As Table 3 shows, their inclusion reduced the canonical correlation between sleep quality and academic performance but for both Motivation and Fatigue included as a potential explanatory variable, the relation between the canonical variates remained significant. However, In Step C, simultaneous inclusion of Motivation and Fatigue in the same model, the canonical correlation between sleep quality and academic performance was reduced by 25%, with the correlation becoming non-significant (see Table 3).

**Table 3:** Statistical unique effects, for potential explanatory variables.

Covariates included	Canonical correlation	Wilks' Lambda	*F*(*df*)	*p*-value
Step 0. No covariates	0.20	0.94	2.07^*^(14,1064)	0.0113
Step 1a. Motivation	0.19	0.95	1.79^*^(14,1056)	0.0348
Step 1b. Fatigue	0.17	0.96	1.72^*^(14,1062)	0.0467
Step 2. Motivation; fatigue	0.15	0.96	1.36(14,1054)	0.1672

^*^p < 0.05.

### 3.7 Question 4

Question #4 assessed Gender and Grade Level as potential statistical moderators of the relations between the PSQI components and Academic Performance (as identified in Question 2, above). For the purposes of this question, a general linear models framework in SAS Proc GLM was used, structured to be parallel to the canonical correlation analysis but with moderators included in the model. The moderator variables were dummy-coded by SAS, using the standard 1, 2 dummy-coding in Proc GLM for 1 df categorical variables. This analysis included Student-reported GPA as the dependent variable, the one Academic Performance variable above the 0.40 cut-off in the canonical correlation analysis. The independent variables included the main effects for the seven PSQI sub-scales as well as the main effects for the moderator variable (Gender, Grade, in separate models), and the interaction terms between the moderator and the three PSQI sub-scales above the 0.40 cutoff. Significant statistical moderator effects were broken down using the Proc GLM Estimate function, which provides estimates for effects at specific points of the moderator. In the present case, this included estimates for males and for females (for significant Gender moderator effects), and estimates for Grade 10 and for Grade 11 (for significant Grade moderator effects).

Two of the three moderator effects for Grade were significant (see Table 4), with none of the three moderator effects for Gender significant (see Table 5). For the Grade X Sleep Duration effect, the break-down analyses (see Table 6) indicated that the effect of Sleep Duration was significant at Grade 11 but not at Grade 10. In contrast for the Grade X Day Dysfunction effect, the breakdown analyses indicated that the effect of Day Dysfunction was significant at Grade 10 but not at Grade 11 (see Table 6).

**Table 4:** Moderator analysis results for grade.

Effect	Beta	SE (β)	*t*-value	*p*-value
Overall Rating	0.02	0.05	0.50	0.6186
Sleep disturbance	−0.03	0.05	−0.72	0.4749
Sleep duration	−0.21	0.06	−3.65^***^	0.0003
Sleep efficiency	0.06	0.05	1.18	0.2371
Sleep latency	0.06	0.05	1.30	0.1930
Day dysfunction	−0.04	0.06	−0.72	0.4744
Medication use	−0.07	0.06	−1.29	0.1989
Grade	0.38	0.09	4.49^****^	0.0001
Grade ^*^ sleep duration	0.25	0.09	2.81^**^	0.0052
Grade ^*^ day dysfunction	−0.20	0.09	−2.29^*^	0.0223
Grade ^*^ medication use	−0.02	0.10	−0.17	0.8653

^*^p < 0.05.

^**^p < 0.01.

^***^p < 0.001.

^****^p < 0.0001.

Student-reported GPA was dependent variable in these analyses.

**Table 5:** Moderator analysis results for gender.

Effect	Beta	SE(β)	*t*-value	*p*-value
Overall Rating	0.00	0.06	0.09	0.9302
Sleep disturbance	0.01	0.05	0.38	0.7022
Sleep duration	−0.09	0.04	−1.38	0.1690
Sleep efficiency	0.07	0.06	1.41	0.1583
Sleep latency	0.06	0.05	1.43	0.1537
Day dysfunction	−0.05	0.04	−0.95	0.3421
Medication use	−0.06	0.06	−0.99	0.3238
Gender	0.36	0.06	4.21^****^	< 0.0001
Gender^*^sleep duration	−0.06	0.09	−0.79	0.4304
Gender^*^day dysfunction	−0.03	0.09	−0.34	0.7328
Gender^*^medication use	−0.05	0.08	−0.57	0.5692

^****^p < 0.0001.

Student-reported GPA was dependent variable in these analyses.

**Table 6:** Moderator breakdown analysis results, for significant moderator effect of grade.

Independent variable	Moderator level	β^a^	SE (β)	*t*-value	*p*-value
Sleep duration	Grade = 10	0.04	0.08	0.50	0.6192
	Grade = 11	−0.21^***^	0.06	−3.65	0.0003
Day dysfunction	Grade = 10	−0.24^***^	0.07	−3.40	0.0007
	Grade = 11	−0.04	0.06	−0.72	0.4744

^a^standardized beta for relation between independent variable and GPA.

^***^p < 0.001.

## 4 Discussion

Before considering the implications of the results of this study, it is important to first note a primary study limitation, which is that the data were cross-sectional. Thus, conclusions regarding causality between various variables cannot be drawn. However, the results do suggest potentially important areas for future research, as insomnia is one of the most prevalent mental health problems worldwide, and an important predictor of impairment in life functioning (American Academy of Sleep Medicine, 2023). During adolescence, a key developmental time period, academic achievement is an important life functioning domain impacted by insomnia. Insomnia itself has multiple components such as sleep efficiency and sleep duration, and understanding how these different components are specifically related to life functioning will be crucial in order that interventions can most efficiently target different components of insomnia. The primary purpose of the present study was to assess relations of the components of insomnia to academic performance among high-school students in Vietnam, a nation where adolescents' academic achievement is strongly emphasized and valued.

### 4.1 Main effect relations between sleep quality and academic performance

Canonical correlation analyses for Question 1 indicated a significant relation between insomnia and academic performance, with the first canonical correlation equal to 0.20. The second canonical correlation was non-significant, indicating that in the present sample there was a single dimension underlying relations between insomnia and academic performance. The signs of the standardized canonical coefficients for the first canonical correlation indicated that within this relation, increased insomnia was associated with decreased academic performance. The standardized canonical coefficients also indicated that three components of the PSQI questionnaire had unique relations (i.e., non-overlapping with other components of the PSQI) to academic performance and were primarily responsible for this relation: Sleep Duration, Day Dysfunction (e.g., trouble staying awake), and Medication Use (for treatment of insomnia). On the PSQI, Sleep Duration represents a component of insomnia whereas Day Dysfunction and Medication Use represent functional impairment associated with insomnia. Thus, Sleep Duration would be seen as the central component of sleep quality and insomnia related to academic performance.

In interpreting these and other findings, it is again important to consider an important study limitation, which is that this study's data were cross-sectional, and therefore do not provide information regarding the direction of causality. Thus, it is possible that within this sample insomnia resulted in reduced academic performance but it is also possible that conversely, low academic performance resulted in increased insomnia, and both directions of causality simultaneously is possible. In addition, third variable explanations (e.g., economic stress causing both insomnia and reduced academic performance) cannot be ruled out. A recent systematic review by Bacaro et al. (2023) of longitudinal research found that insomnia was longitudinally predictive of reduced academic performance, but that the other direction of causality also can occur. So it will be important for future research focusing on specific components of insomnia to use longitudinal designs to provide information regarding direction of causality.

In considering the implications of the relation between sleep quality and insomnia, and academic performance as discussed below, it is important to consider that the size of the relation between these constructs was small, *r* = 0.20. However, even small effect sizes can have importance if linked to clinical interventions and outcomes (Funder and Ozer, 2019; Prentice and Miller, 1992). Further, evaluation of the *incremental* value of a relation requires consideration of the broader structural context of the outcome in relation to other predictors (Götz et al., 2022). Overall, academic performance is substantially determined by stable, relatively unmodifiable predictors such as intelligence and prior academic achievement (Roth et al., 2015). Within this context, even a relatively small potential effect (in the present case, from insomnia) can carry practical significance, if this factor is modifiable (Funder and Ozer, 2019). In addition, as Götz et al. (2022) have demonstrated, small incremental effects on continuous performance outcomes can accumulate over time, yielding meaningful population-level impacts across multi-year academic trajectories. Thus, it will be important for future research to develop efficient methods for utilizing these relations, if they are supported by longitudinal research.

#### 4.1.1 Sleep duration

Current findings provide important hypotheses and directions for future research. If future experimental and longitudinal research confirms that sleep duration has a specific (i.e., beyond other components of insomnia) causal influence on academic performance, it will have important implications for treatment, at least in regard to adolescents in countries similar to Vietnam where academic achievement is particularly highly valued. A number of different behavioral and psychological interventions have been found to be effective in treating insomnia (Galgut et al., 2025), but with different profiles of strengths and disadvantages. For instance, Sleep Restriction Therapy (designed to improve sleep efficiency) and Sleep Extension Therapy (designed to increase sleep duration) both have been shown to be effective treatments for insomnia (Arnal et al., 2015; Wei et al., 2025), but with different strengths and disadvantages (e.g., Gruber et al., 2012). For example, Sleep Restriction Therapy can significantly reduce insomnia but is associated with increased treatment dropout and reduced treatment compliance compared to other treatments (Gruber et al., 2012). The fact that sleep duration—specifically targeted by Sleep Extension Therapy—and not sleep efficiency was the primary symptom component of insomnia related to academic performance suggests that future experimental research to confirm these relations will be important, in order to determine the utility of Sleep Extension Therapy as a useful treatment for this population. Thus, an important area for future research will be to confirm these present findings longitudinally and with experimental research.

#### 4.1.2 Day dysfunction

The two functional impairment components of the PSQI, Day Dysfunction and Medication Use, both also showed unique relations to academic performance. “Functional impairment” is defined as clinically significant distress or impairment in social, occupational, or other important areas of functioning (American Psychiatric Association, 2022). Functional impairment itself has been differentiated into (a) proximal (process-related) functional impairment vs. (b) distal (outcome-related) functional impairment (Krieger, 2008). In the present case, Day Dysfunction (trouble staying awake during the day; lacking enthusiasm to get things done) would represent proximal functional impairment whereas reduced academic performance (the outcome in the present study) would represent distal functional impairment. This suggests that, given that day dysfunction showed a substantively meaningful unique relation to academic performance, the components of day dysfunction might be important secondary targets for intervention, if the initial intervention focused on increasing sleep duration does not result in the desired outcomes (i.e., increased academic performance). If future longitudinal research confirms these findings, this also suggests that the day dysfunction components could be used as intermediate outcomes to assess the effectiveness of the intervention vis-à-vis improving academic performance. Such a conclusion, of course, is dependent on future research identifying a causal relation.

#### 4.1.3 Medication use

The third and final component of the PSQI that had a unique relation with academic performance was the sub-scale Medication Use. Higher levels of medication use were associated with lower levels of academic performance. This initially might seem counter-intuitive, as randomized clinical trials evaluating the effects of medication on insomnia and sleep functioning generally have found that medication use results in reduced insomnia (e.g., De Crescenzo et al., 2022). However, in the present study medication use was not randomized. Thus, it is possible that participants with more severe insomnia had an increased likelihood of using medication as compared to participants with less severe insomnia, with their more severe insomnia associated with higher impairment in GPA, though future longitudinal research will need to confirm such findings. In addition, sleep medication can have significant side-effects (e.g., Sateia et al., 2017), and although the medication may increase sleep duration this may come at the cost of sleep quality, potentially resulting in life impairment including in academic performance. Further, as Jacobs (2009) had noted, sleep medication often only increases sleep duration for a relatively short amount of time, and this additional sleep generally is light sleep, with the medication making it difficult to enter deep sleep and dream sleep—the two most important stages of sleep (Jacobs, 2009). This can increase fatigue and impair the brain's ability to process information during the day (Jacobs, 2009). Such findings support the general perspective of the field that medication should be approached with caution, even in regard to non-prescription medications (Sateia et al., 2017). In interpreting results involving Medication Use, it is important to note that the rate of medication use was relatively low in the present sample, less than 10%. Thus, it will be important for future research to confirm the present finding.

#### 4.1.4 Fatigue and motivation

In addition to the PSQI, the study assessed fatigue and motivation as potential explanatory variables for relations between insomnia and academic performance. Results of analyses for Question #3 indicated that when these factors were both included in the canonical correlation model, the relation between the PSQI and academic performance became non-significant. This supports the finding that these factors may be statistical explanatory variables, although full confirmation of such an interpretation will require future longitudinal research. If this finding is confirmed with future longitudinal research, it does suggest that the two factors should be clinically monitored. If the client is not showing expected improvement, then it may be useful for treatment to target additional areas such as, for instance, low motivation related to insomnia, perhaps using insomnia-related behavioral activation (Manber et al., 2011) as part of the intervention.

### 4.2 Moderator effects

Finally, the last set of analyses (Question 4) assessed whether the unique statistical relations identified in the primary analyses differed as a function of Gender or Grade. All of the moderator effects with Gender were non-significant, indicating that the unique effects of the PSQI components did not vary as a function of the participant's gender. However, in regard to Grade, (a) the negative relation between Day Dysfunction and Student-reported GPA was significantly larger for Grade 10 than Grade 11, but (b) the negative relation between Sleep Duration and Student-reported GPA was significantly larger for Grade 11 than for Grade 10. In interpreting these results, it is important to consider the structure and expectations in Vietnamese high-schools. Although in the present analyses “Grade” represented a single grade difference (10^th^ vs. 11^th^), within Vietnam this represents a significant distinction. In Vietnam, high-school starts with Grade 10, with a major focus of Grade 10 on supporting students in transitioning to high-school, with its different structure, expectations, etc. (Dương et al., 2021). In contrast, a major focus of Grade 11 is on preparation for college applications and exams, with significantly increased expectations and stress (Dương et al., 2021).

Thus, one possible reason for this statistically significant moderator effect is that Grade 11 students may be more likely to “push through” fatigue and low motivation (Day Dysfunction) to continue studying than Grade 10 students, as academic success becomes essentially fundamental in Grade 11. This could result in the observed interaction where the relations between fatigue and low motivation with academic success are smaller for 11^th^ Grade students. However, the functional impairment associated with insomnia extends beyond fatigue and motivation, to direct neurological impairment in working memory, executive functioning, and other such processes (Fortier-Brochu and Morin, 2014; Wardle-Pinkston et al., 2019) less reduced by determination. The higher expectations for 11^th^ Grade students in combination with effects of insomnia non-responsive to determination thus might hypothetically result in the observed interaction, where relations between insomnia and academic performance are larger for 11^th^ grade students because of the increased expectations. If these interpretations are confirmed through additional research including longitudinal research, it would suggest that for younger high school students, insomnia interventions should include a direct focus on reducing related functional impairment. For older high school students, it further supports the critical importance of emphasizing increasing sleep duration, to directly reduce cognitive impairment associated with insomnia (Boergers et al., 2014).

### 4.3 Dependent variables

An important result within these findings was that for the second canonical variate (Academic Performance), only Student-reported GPA had a unique relation. In the present dataset, Student-reported GPA was based on the adolescent's report of their number-based GPA whereas Subjective GPA was based on their subjective evaluation ranging from Unsatisfactory (coded as 1) to Excellent coded (coded as 4). There are several implications of this finding. First, within the various study limitations as discussed in the 4.4 Limitations section (below), it suggests that insomnia's relation to academic performance may be not be so much to the student's subjective evaluation of their performance (Subjective Academic Performance) since Subjective Academic Performance was not a part of Academic Performance canonical variate. That is, if this result were due to the students' subjective evaluations of their performance, it might be expected that Subjective Academic Performance would have also been a component of the Academic Performance canonical variate. Second, if this finding is confirmed with future research, it supports the importance of insomnia and impaired sleep quality for actually impacting on academic performance, not simply the students' perceptions of their importance.

### 4.4 Limitations

An important limitation of the present study, as noted above, is cross-sectional nature of the research design, which means that temporal precedence and causality cannot be evaluated. In addition, as discussed above in 4.3 Dependent Variables, another limitation was that all data were based on adolescent self-report, including students' GPA. Thus, it is possible that the relation between insomnia and academic performance was a consequence of self-report bias rather than an actual relation between the two constructs. That is, it is possible that persons with higher levels of insomnia may have a more negative biased perspective regarding themselves, resulting in more negative reports of academic performance. This could result in the observed relation. However, the fact that Subjective Academic Performance was not part of the canonical relation between insomnia and academic performance (see Table 2) argues against such an interpretation, though it will be important for future research to include actual grades from the school.

Another limitation is that in order to ensure complete confidentiality and privacy for the student participants, identifying information including region, school, and classroom were not collected. Thus, it was not possible to control for clustering across schools, etc. Also, because the participants were students, information regarding family socioeconomic status (e.g., parental income) was not able to be collected. Thus, it is possible that there is unmeasured confounding among the analyses, which was not able to be controlled due to a lack of specific variables. Finally, although data validity analyses were conducted (e.g., identifying multivariate outliers), such analyses cannot absolutely confirm the validity of the data.

### 4.5 Conclusions

Insomnia is a prevalent problem that can have significant effects on life functioning. Results of the present study suggest longitudinal research assessing effects of specific components of insomnia in regard to causality will be important. If confirmed by this research, results suggest that to most efficiently reduce the effects of insomnia on academic performance in countries such as Vietnam where academic success is particularly highly value, it may be important for interventions to focus on enhancing sleep duration. If such conclusions are supported by future longitudinal research, treatments such as sleep extension therapy may be useful in this regard. This may be particularly true for older students, such as those in Grade 11. Future research using longitudinal designs and teacher-report of academic performance will be critical in order to confirm these conclusions.

## Data Availability

Data analyzed in this study are available for study replication purposes upon reasonable request, from Bahr Weiss at Bahr.Weiss@Vanderblt.edu.
